# A biopsychosocial model of MDMA-assisted therapy in application: Dyadic One Session Treatment for specific phobia

**DOI:** 10.3389/fpsyt.2025.1665770

**Published:** 2025-09-05

**Authors:** Nicholas J. Ahari, Gregory A. Fonzo

**Affiliations:** ^1^ Department of Counseling and Clinical Psychology, Teachers College, Columbia University, New York, NY, United States; ^2^ Department of Psychiatry and Behavioral Sciences, Charmine & Gordon McGill Center for Psychedelic Research and Therapy, University of Texas at Austin Dell Medical School, Austin, TX, United States

**Keywords:** MDMA, PTSD, fear, phobias, biopsychosocial, exposure, transdiagnostic, psychedelic

## Abstract

3,4-methelenedioxymethamphetamine (MDMA) can be effective in treating posttraumatic stress disorder (PTSD) in controlled trials, potentially secondary to MDMA’s effects on neural circuits implicated in fear and reward. Although anxiety, stress, and fear-based disorders involve maladaptation of the neural circuits processing fear, threat, and reward, no studies have tested MDMA’s therapeutic efficacy on specific phobias. This article proposes a naturalistic biopsychosocial model of MDMA assisted therapy (MDMA-AT) informed by the neurobiological mechanisms of MDMA and the theoretical models of Emotional Processing Theory (EPT), inhibitory learning, and cognitive behavioral interpersonal theory (CBIT) to inform transdiagnostic treatments for anxiety, stress, and fear-based disorders. As a fear-based disorder with a circumscribed focus, we apply the biopsychosocial model to propose a novel MDMA-assisted Dyadic One Session Treatment (DOST) model for spider phobia, one of the most common animal phobias. Specific phobias such as spider phobia offer a straightforward naturalistic model to test the effects of MDMA on normalizing approach behavior, avoidance behavior, and neural circuit function. We hypothesize that the neurobiological and prosocial effects of MDMA can promote enhanced emotional processing and inhibitory learning of phobic stimuli during exposure exercises to create more adaptive associations that lead to increases in approach behavior and reductions in spider phobia symptomatology. Such a model may spur greater thought towards integration of evidence-based exposure therapies (ETs) designed to optimally capitalize upon the pharmacological effects of MDMA and other psychedelic compounds to treat fear-based mental health conditions.

## 1 Introduction

**Figure 1:**
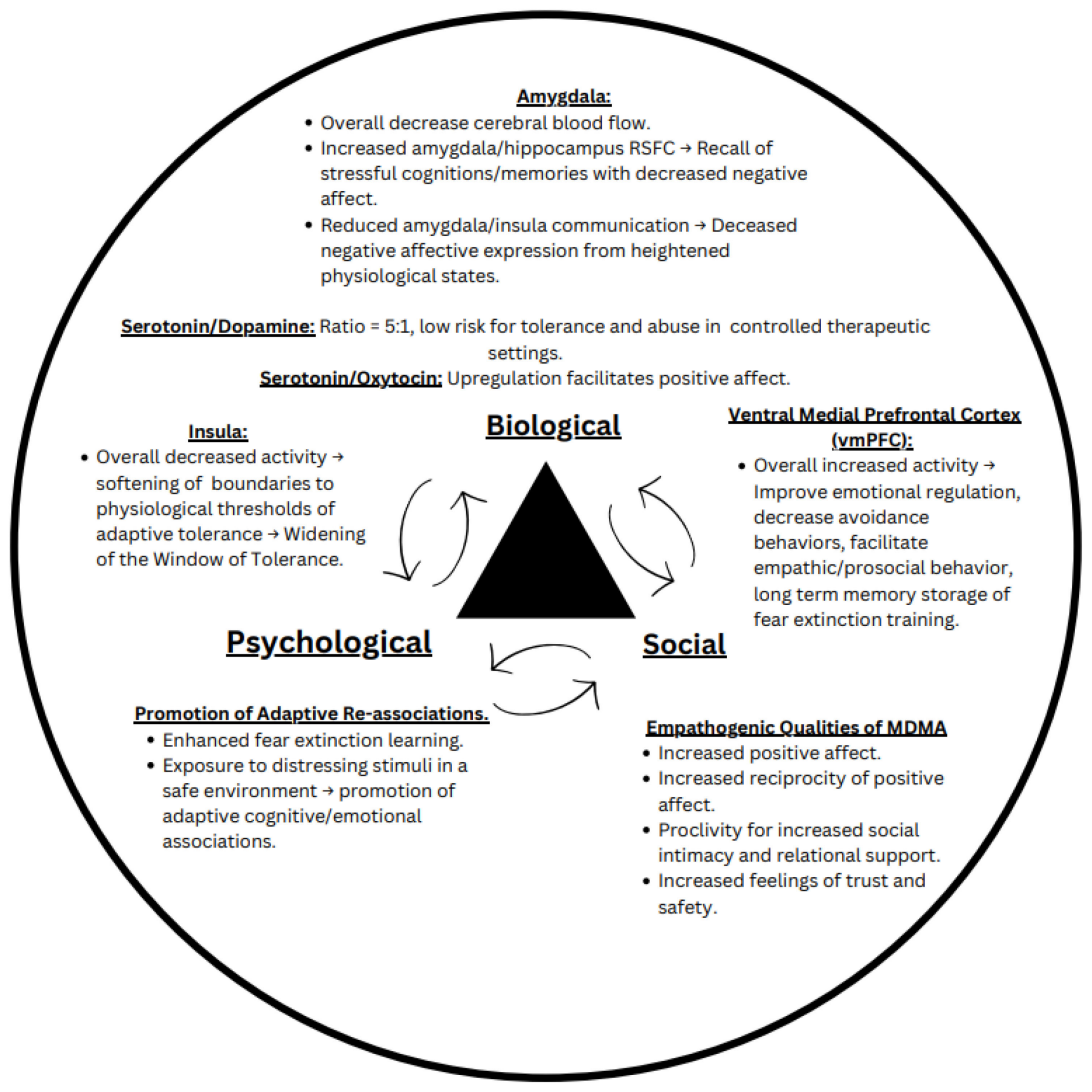
The biopsychosocial model of MDMA assisted therapy. This proposed model outlines the known effects MDMA has on neurological circuits implicated in fear and reward processing (biological component), ability to promote fear extinction and cognitive/emotional processing (psychological component), and proclivity to increase prosocial behavior and positive affect (social component).

It has been proposed that the pharmacological effects of MDMA, a substituted phenethylamine, fosters engagement in therapeutic settings by enhancing emotional regulation and decreasing avoidance behavior (1). MDMA, which has unique subjective effects and mechanism of action that distinguish it from classic psychedelics, is often called an entactogen or “empathogen” for its capacity to promote prosocial emotions, even increasing social approach behavior in asocial animals like octopi (2). It furthermore seems to attenuate processing and perception of threat and negative emotionality, such as reducing recognition of negative facial expressions (3–5). Moreover, research has begun highlighting the importance of studying MDMA on a neurobiological level to advance understanding of basic prosocial psychological processes (6). Despite recent research illuminating the biological, psychological, and social effects of MDMA, there is no comprehensive biopsychosocial model that can inform optimal delivery of MDMA-assisted therapies (MDMA-ATs). Here, we propose a biopsychosocial model of MDMA-ATs (**Figure 1**) that highlights the bidirectional relationship between MDMA’s (a) dynamic effects on neural circuitry, (b) promotion of positive cognitive/emotional associations, and (c) propensity for reciprocal prosocial behavior.

## 2 Background

The proposed biopsychosocial model is used to inform the design of a novel exposure therapy (ET) for spider phobia. However, this model is transdiagnostically relevant to all diagnoses under the umbrella of anxiety, stress, and fear-based disorders treated with evidence based ETs. We also note that ongoing research is examining MDMA as an adjunct to ET for PTSD in the form of Massed Exposure Therapy Enhanced with MDMA for PTSD (METEMP), which highlights another important thread of research along this vein (7, 8). Spider phobia was chosen as an exemplar of a relatively uncomplicated, fear-based disorder to illustrate MDMA’s effects on neural circuits implicated in fear/threat that are commonly implicated across anxiety, stress, and fear-related disorders including PTSD (9–12). Although specific phobias generally respond well to ET, 25-30% of individuals do not derive adequate therapeutic benefit (13). So, although MDMA-AT for specific phobia may not be a first-line treatment option (assuming eventual FDA approval of MDMA-AT), the translational relevance of the experimental model of fear conditioning and extinction to simple phobias offers a useful clinical framework to begin studying the optimization of MDMA-AT protocols with ET and/or dyadic interventions. Such protocols may be further optimized for the treatment of more complex and treatment-resistant diagnoses, such as PTSD and/or social anxiety disorder (SAD). Below, the theoretical structure to the biopsychosocial model is outlined, followed by the neurobiological mechanisms of MDMA and fear extinction. After, the current state of knowledge for specific phobias is described, along with current treatment modalities and their limitations. The biopsychosocial model is then applied to a proposed novel Dyadic One Session Treatment (DOST) model for specific phobias with a proposed 2x2 factorial analysis to determine if fear extinction learning can be enhanced using dyadic models and/or MDMA augmentation.

### 2.1 Theoretical structure

The biopsychosocial model draws from the frameworks of Emotional Processing Theory (EPT), inhibitory learning, and cognitive behavioral interpersonal theory (CBIT) to illustrate how MDMA’s effects on emotional processing and prosocial behavior can be integrated into a single theoretical framework, as described in the following sections.

#### 2.1.1 Emotional Processing Theory and inhibitory learning

EPT outlines how emotionally salient cognitive networks are developed, modified, and maintained (14, 15). Consistent with Pavlovian associative learning, EPT highlights initiation of emotional structures in response to stimuli and informs many evidence-based ETs (15). For anxiety disorders like specific phobia, emotional structures target distressing stimuli by developing predictive stimulus-response relationships that constitute cognitive networks containing information on stimuli and their physiological, behavioral, and affective responses (15, 16), like the sight of a spider eliciting a panic attack (17). Components of emotional structures include two unconditioned stimuli (US) paired in an emotionally evocative way, creating an aversive conditioned stimulus (CS+) and conditioned response (CR+). For spider phobia, the CS+ of spiders elicits the CR+ of irrational fear triggered by the thought or sight of spiders with overgeneralized cognitive structures like “spiders are universally dangerous and should be rightfully feared.” This process can facilitate avoidance behaviors, behavioral rigidity, exaggerated physiological responses, and impairments in threat appraisal of serious detriment to healthy functioning (18). EPT implies aversive experiences are integral to developing pathological fear, with research indicating direct aversive experiences are likely the most common etiological root of anxiety disorders (17). Note that the primary etiology of spider phobia is a point of contention which may be evolutionarily based (19).

Consistent with Pavlovian learning, inhibitory learning is considered the primary mechanism of long-term avoidance behavior, fear, and anxiety reductions (16, 20, 21). Inhibitory learning does not entail an erasure of conditioned fears during exposure, but instead introducing new information during extinction training, so that the aversive CS develops a nonpredictive relationship with the aversive US (16, 20). In essence, within an inhibitory learning approach, the aversive CS-US relationship remains intact, along with a new inhibitory CS-alternative US relationship, that is not dependent on expression of fear within extinction training (16). Although it has long been argued that elicitation of genuine fear responses and corresponding physiological arousal are integral to fear extinction learning, others have found that level of fear during exposure is not paramount to adaptive learning, and that the emphasis should rather be placed on optimizing the efficiency and resilience of inhibition learning through time and context (16). It is believed that targeting inhibitory learning systems can help optimize exposure therapies, as anxious individuals often exhibit learning deficits in this area (20). Some strategies proposed to maximize inhibitory learning for extinction include designing exposures that optimize violation of expectancies for pathologically fearful outcomes, introducing novel information during retrieval of aversive memories or cognitions to change them, and exposure to multiple excitatory stimuli during extinction training (16, 20). The latter is derived from error correction model (22), positing that contradictions between the summed associative strength of all present stimuli, and each of their saliences, without reinforcing the US, determines the effectiveness of extinction training.

Effective use of ETs necessitates exposure to an aversive CS+ in a safe environment that runs contradictory to the fearful association, allowing for new learning to occur in response to a neutral outcome (15, 18, 23). Those with specific phobia can then realize their fear is unrealistic, tolerable, and temporary (15). With avoidance being fundamental to the maintenance of anxiety and stress disorders, it is argued clients must fully engage in exposure processes (18). We hypothesize that MDMA’s ability to promote adaptive neurological processing of feared stimuli may enhance the replacement of maladaptive associations constituent of emotional structures with more adaptive information by enhancing engagement with feared stimuli during dyadic ET and facilitating effective inhibitory learning when implemented in a dyadic protocol.

#### 2.1.2 Cognitive-behavior interpersonal theory

Cognitive-behavior interpersonal theory (CBIT), which was developed to describe PTSD, posits there are bidirectional emotional, cognitive, and behavioral factors within relationships helping prevent, maintain, or exacerbate PTSD symptomatology (24, 25). CBIT breaks down how the various symptom clusters of PTSD negatively affect interpersonal relationships. Moreover, CBIT postulates how those close to the traumatized individual often unintentionally collude with PTSD avoidance symptoms to mitigate distress from the traumatized individual in what is called “behavioral accommodation,” contributing to the persistence of PTSD symptoms (24), which has been observed in parents of children with specific phobia (26), and more generally across fear-based disorders (27). Thus, although CBIT was developed in the context of PTSD, the theoretical tenets are broadly applicable across various fear and stress-based disorders.

CBIT underlies the treatment modality of cognitive-behavioral conjoint therapy (CBCT) for PTSD. There is a small body of literature on CBCT for PTSD, with systematic reviews revealing most studies utilize uncontrolled designs, and no studies utilize active control groups (28). However, in almost all studies CBCT yields significant improvements in patient PTSD symptomatology and relationship satisfaction (28). A randomized control trial of CBCT for PTSD resulted in significant reductions of PTSD symptomatology, relationship satisfaction, and showed one of the largest beneficial post treatment effect sizes to date when compared to a wait-list control group (29). However, these findings are limited by a small sample size. More recently, the largest sample to date (n = 113 couples) with an intention-to-treat design of military veterans from a variety of service eras with PTSD or subthreshold PTSD, was the first study to track PTSD symptomatology throughout the 15-session treatment schedule (25). Findings indicated significant reduction in self-reported PTSD symptoms with effects sizes like those in previous studies involving veteran populations (d = -0.69), and significant increases in relationship happiness for both veterans and their partners (25). Notably, 34.8% of veterans met criteria for recovery and 27.7% met criteria for reliably improved (25). However, this study did not have a control group and exhibited a high dropout rate, highlighting the importance of future studies using individual treatments as active control groups while investigating ways to enhance treatment adherence (25). Taken together, these findings suggest that therapeutically capitalizing on interpersonal dynamics in the context of fear and stress-based disorders, as suggested by CBIT and epitomized by CBCT, may be an effective method for promoting recovery.

#### 2.1.3 Possible psychotherapeutic models for MDMA-AT

MDMA’s empathogenic qualities can reciprocally amplify prosocial behavior between individuals, making it suitable for dyadic cognitive behavioral treatment models (30–32). Here, we detail the current state of literature surrounding CBCT informed dyadic models of MDMA-AT for PTSD, to highlight how the biopsychosocial model can be used from a transdiagnostic perspective.

Dyadic MDMA-AT has historically been used in couples therapy throughout the 1970s and 1980s (30, 31, 33). In more recent years, a small open-label pilot study for dyadic MDMA-AT for PTSD yielded large improvements for PTSD symptomatology and its comorbidities (30). Another study on dyadic MDMA assisted couples therapy for PTSD demonstrated how improved psychosocial functioning resulted in enhanced relational engagement (31). A case study of a female military veteran engaging in an open-label clinical trial of MDMA assisted brief cognitive behavioral conjoint therapy (bCBCT) for PTSD, also experienced significant reductions in PTSD symptoms and enhanced relationship satisfaction with her intimate partner at 6 month follow ups (34). However, all studies had very small sample sizes with no placebo control group, serving as motivation for further investigation into dyadic MDMA-ATs with larger sample sizes and double-blind placebo-controlled designs. We contend that, with a biopsychosocial approach capitalizing on the fear/threat attenuating and empathogenic qualities of MDMA, such dyadic therapeutic models could enable optimal emotional processing for ETs treating anxiety disorders such as specific phobias.

### 2.2 Therapeutic mechanisms of MDMA

Considering the theoretical frameworks of the biopsychosocial model for MDMA-AT, it is important to understand the neurobiological changes one experiences post MDMA administration. Regardless of pharmacological intervention, understanding the neurobiological mechanisms of fear and its extinction can lead to improved treatment outcomes in cognitive behavioral therapeutic models (18). The following sections outline how MDMA enhances fear extinction from a metaplastic perspective, before detailing the current knowledge of MDMA’s effects on neural circuitry involved in fear. “Metaplasticity” refers to higher order, multidirectional neuroplastic actions of change, occurring when synaptic plasticity modulates further synaptic plasticity, and leaves lasting impacts on synaptic function (35, 36). In essence, metaplasticity refers to dynamic regulation of the extent to which plasticity can occur. Metaplasticity can occur, but is not limited to, downstream of classic and non-classic psychedelic administration (37), which differentiates psychedelics from drugs of abuse such as cocaine and alcohol that exhibit more bidirectional hyper- or hypo-plastic synaptic activity (36). Metaplasticity is also believed to contribute to MDMA’s lasting rehabilitative qualities with minimal dosing sessions (38).

#### 2.2.1 MDMA and fear extinction

Fear extinction is defined as decreased conditioned fear responses to previously feared stimuli after repeated presentation without aversive outcomes (39, 40). Repeated lack of aversive outcomes is thought to promote learning through the formation of new associations with feared stimuli, eventually reducing fear responses (1).

There is a growing notion that MDMA’s therapeutic effects may derive from its ability to widen the Window of Tolerance, the zone of optimal physiological arousal for functional engagement with the world, that is unique to everyone (23, 31, 41–43). From a cognitive perspective, research suggests MDMA’s effects on fear extinction may promote more adaptive associations to previously feared stimuli, with MDMA’s effects on fear-related circuitry helping to explain improvements in fear extinction. Such circuit alterations from MDMA include downregulation of left and right amygdala activity (1, 44, 45), upregulation in resting state functional connectivity (RSFC) between the amygdala and hippocampus (43, 44), downregulation of insular activity while maintaining connectivity to the amygdala and hippocampus (12, 46), and upregulation of the ventromedial prefrontal cortex (vmPFC) (1, 45). Cumulatively, MDMA’s effects are theorized to allow for recall of typically distressing memories/cognitions in a state of heightened tolerance to intensely visceral experiences normally associated with negative affect (41, 47).

In the simplest of terms, phobic associations are overgeneralized fear associations in neutral contexts that impair healthy functioning. Anxiety driven maladaptive associations implicate both the medial prefrontal cortex (mPFC) and the basolateral amygdala (BLA), which both play a role in fear extinction processes and inhibit extinction learning when ablated in laboratory settings (39, 40, 48, 49). It has been asserted that neuroplastic alterations in such regions, along with the hippocampus, are integral to extinction learning, all of which exhibit alterations post MDMA administration (40, 50–53).

In rodent studies, MDMA administered 30 minutes prior to extinction training significantly improved fear extinction long term measured by decreases in conditioned freezing, persisting after presentation of feared objects in novel contexts over a week later. This is something most pharmacological modalities fail to accomplish, particularly with a single dose (54). Rats who receive MDMA during fear reconsolidation also show later onset and persistently dampened conditioned fear responses (55). Moreover, mice chronically administered 5-HT transporter (5-HTT) inhibitors for 22 days prior to MDMA assisted extinction training did not display any reductions in conditioned freezing relative to those who received MDMA without chronic administration of such inhibitors (56). However, inhibition of norepinephrine and dopamine did not interfere with fear extinction, indicating serotonin receptors as integral for MDMA assisted fear extinction (56). Of note, MDMA increases serotonin synaptic activity at rates five times higher than dopamine (44).

Studies in humans have also demonstrated MDMA enhancement of fear extinction. One study using classic Pavlovian conditioning had 34 adults engage in extinction training 2 hours and 24 hours post MDMA administration (46). More participants in the MDMA group retained fear extinction learning relative to the placebo group (46). However, within-session extinction learning was not improved (46). Another study in 30 healthy male subjects administered MDMA (vs. placebo control) after fear conditioning and two hours prior to extinction learning, with those receiving MDMA showing reduced fear responses in the early phase of extinction training compared to the placebo group. The effect persisted in the recall phase 22 hours post extinction learning, as measured by skin conductance response (4). A negative correlation was also found between the intensity of MDMA’s acute effects during extinction recall and discrimination between the “safety stimulus” (CS-) and the CS+ during extinction recall (4).

#### 2.2.2 MDMA’s effects on neural circuit function

Considering the research on MDMA and fear extinction, it is also believed that MDMA’s therapeutic utility may be attributed to its ability to increase feelings of trust and safety (46). Such enhancements in safety and trust may be secondary to inhibition of neural circuits implicated in fear and threat, and possibly, also, an upregulation of circuits involved in reward/positive valence processes and social cognition. Below, we outline key neurobiological structures implicated in MDMA’s ability to promote prosocial emotions and enhance fear extinction.

##### 2.2.2.1 Medial/ventromedial prefrontal cortex

The medial prefrontal cortex (mPFC) reciprocally projects information to the amygdala and is known to facilitate dampening of conditioned fear responses (18, 57). Its ventral portion, the ventromedial prefrontal cortex (vmPFC), shares bidirectional connections to areas of the brain implicated in threat and fear-based learning, such as the amygdala and hippocampus (58). Amongst neural circuits implicated in fear and reward processing, the vmPFC specifically functions to encode afferent sensory information into long term memory storage (59). Cumulatively, the vmPFC is thought to bring affective meaning through “organism-wide emotional behavior” (58, 59). Essentially, the vmPFC assigns emotional value to sensory stimuli, facilitating recognition of emotional associations for decision making purposes like risk evaluation and learned probabilistic reasoning from negative or positive feedback (58).

Multiple lines of research suggesting the vmPFC provides top-down regulatory influence over the amygdala to relieve states of fear in both rodents and humans (60, 61). In fact, vmPFC activation can facilitate successful fear extinction, and damage to the vmPFC leads to impairments to the retention of extinction learning (62). For instance, rats with complete vmPFC lesions were indistinguishable from control rats who received no extinction learning two days after fear acquisition and extinction training, with an 86% fear recovery rate (63), indicating that the vmPFC may contribute to long term recall of learned safety post extinction training (18, 63), i.e. extinction retention. However, lesions sparing damage to the caudal infralimbic (IL) cortex, a portion of the vmPFC, had no effect on spontaneous recovery of fear responses, suggesting that portions of the vmPFC, especially the IL, are integral to recalling fear extinction learning (63). Additional evidence clearly indicates a regulatory role of the vmPFC over the amygdala. For example, rodents demonstrated diminished acquisition of fear when pairing fear conditioning with vmPFC stimulation (62). Experimentally manipulating mPFC neurons to fire has also yielded a negative correlation (r = -0.73) with fear recovery in rats post Pavlovian fear extinction training, which were inactive during fear conditioning (64).

Apropos to the mPFC’s role in fear inhibition and extinction learning, fear and stress-based disorders in humans, such as PTSD, also show abnormalities in medial prefrontal function. PTSD is marked by hypersensitive amygdala responses to trauma relevant reminders with impairments on top-down inhibition to the amygdala from the vmPFC, a prerequisite neurological function of fear extinction (1, 65–67). For instance, a study using positron emission tomography (PET) found increases in regional Cerebral Blood Flow (rCBF) to the medial frontal gyrus negatively correlated with rCBF to the left amygdala, and PTSD symptom severity correlated positively with rCBF to the amygdala and negatively with rCBF to the medial frontal gyrus in Vietnam war veterans when recalling their own traumatic events (18, 68).

MDMA in fact, bilaterally upregulates rCBF to the vmPFC in humans when given a simple Continuous Performance Test (CPT). Although this is an emotion-absent psychological process, upregulated rCBF in the vmPFC, more generally, may be one mechanism for improved emotional regulation and decreased avoidance behaviors (1, 45). This may be one substrate mediating prosocial behavior post MDMA administration, as those with lower levels of vmPFC activity and/or vmPFC damage exhibit more egocentric and antisocial behavior (58).

##### 2.2.2.2 Amygdala

The amygdala, an evolutionarily primitive brain region, is integral to the fear response. As a part of the limbic system, the amygdala is a small cluster of nuclei within the medial temporal lobes of each hemisphere that is critically involved in the detection of salient environmental stimuli, affective memory processing, and expression of emotion (40). Amygdala hypersensitivity has been found in those with high trait anxiety that impedes extinction learning (69). Amygdala dysfunction is also considered a central pathophysiological facet in PTSD, possibly mediating elevated threat appraisal often seen in the disorder (70, 71). Moreover, amygdala hyperactivity is observed in SAD and specific phobias such as arachnophobia (72–78). Initial small studies in healthy human subjects have shown that MDMA can reduce one’s subjective fear response, which is correlated with reduced left and right amygdala activation (41, 44, 45, 79), such as findings in which MDMA attenuated left amygdala responses to angry faces while undergoing an fMRI during peak drug effects (80).

However, despite attenuating activity in the amygdala, MDMA has also been shown to increase resting state functional connectivity (RSFC) between the amygdala and hippocampus, which is associated with positive affect, while reducing RSFC between the amygdala and insula (44, 79). Measures of spontaneous neurological changes post MDMA administration through functional magnetic resonance imaging (fMRI) found that increased RSFC between the amygdala and hippocampus correlated with strong subjective effects from MDMA, despite overall decreased cerebral blood flow (CBF) in these same regions (44). Such changes may provide a neurobiological signature of MDMA’s therapeutic effects on disorders such as PTSD, which may allow for adaptive reprocessing of traumatic memories (potentially mediated by amygdala-hippocampal interactions) while tolerating physiological reactivity (potentially mediated through changes in amygdala-insula interactions; see section below for the role of the insula in representing subjective physiological states) (46).

##### 2.2.2.3 Insula

There is substantial evidence to support the role of the insular cortex in interoception of visceral sensations, sometimes referred to as the “viscerosensory cortex,” (10, 81–84). Interoception is considered, in its most basic form, awareness of one’s inner emotions and physiological state (71, 85, 86). Interoception and the insular cortex are key components of the threat appraisal and fear-responsive neural circuitry (71). MDMA administration has been shown to result in decreased RSFC and rCBF in the insular cortex (12, 45), also potentially mediating MDMA’s anxiolytic effects (79). The insula connects internal physiological reactions to perceptions of our surroundings, is active during tasks that engage visceral autonomic sensations (83), and sends efferent projections to the peripheral nervous system (83). fMRI studies investigating mechanisms of interoception and emotional awareness found that the right anterior insula (rAI) may serve as a key hub for explicit subjective awareness (86). In humans, local gray matter in the right anterior opercular region, a thin layer of gray matter covering the insula, were positively correlated with interoceptive accuracy of awareness to one’s own heartbeat (R = .77), and general activity in the rAI and opercular region was positively correlated with performance of this same task (R = .62) (87).

Meta-analyses have cited hyperactivity of the insula across fear-based disorders, including specific phobias, SAD, and PTSD (9, 10, 12). The insular region is believed to play a role in propensity for anxiety by facilitating exaggerated anticipations of stressful bodily conditions (88). In fact, correlations have been observed in decreased connectivity between the right insula and bilateral dorsal-lateral prefrontal cortex (dlPFC) post MDMA administration and greater baseline trait anxiety (R= .61) (12).

Moreover, fear extinction has been facilitated by insular cortex inhibition for mice with extreme low and high levels of fear, rather than intermediate levels of conditioned fear (10), arguing that the insular cortex serves to maintain emotional homeostasis by detecting physiological deviations from pre-established levels of adaptive functioning, a hallmark function of interception (10, 89). Theoretically, this may indicate the insula’s ability to detect thresholds of physiological tolerance, and after MDMA administration, expand this Window of Tolerance by decreasing insular activity.

#### 2.2.3 Specific phobias

Considering MDMA’s ability to promote fear extinction through effects on fear-responsive circuitry as well as the translational relevance of fear conditioning, extinction, and inhibitory learning paradigms common to ETs used in clinical practice, we propose leveraging this joint knowledge base to design and test novel MDMA-assisted adaptations of evidence based ETs. Simple phobias are useful experimental and clinical models for relatively uncomplicated fear-based disorders with circumscribed foci of fear and distress. This clinical condition and its treatment also exemplify the potential explanatory power of fear extinction and inhibitory learning processes theoretically tapped by ETs to facilitate mechanistic inference on how MDMA’s neurobiological effects might alter these processes to promote enhanced therapeutic outcomes. Specifically, understanding MDMA’s ability to assist treatment for relatively uncomplicated, fear-based conditions such as specific phobias can help advance knowledge in designing and applying MDMA-ATs for more complex clinical conditions also characterized by pathological fear, amongst symptoms in other domains, such as PTSD.

Phobias are marked by intense and irrational fear in reaction to specific stimuli, with prevalence rates around 20% in the general population, resulting in avoidance behaviors directed at the feared object or situation that maintain phobic symptomatology and are often comorbid with a variety of anxiety disorders (90–92). Of note, avoidance behaviors are also a core diagnostic criterion of PTSD according to the DSM-5-TR.

Spiders, rodents, and snakes are the most feared stimuli commonly encountered in everyday life, with women having significantly higher rates than men (19, 91). Arachnophobia is seen as arguably the most common specific phobia, defined by intense fear of arachnids including spiders (90, 92). Such phobic reactions can come from simply thinking of spiders, seeing a picture of spiders, and entering a space where spiders have been seen before (92, 93).

Despite the burden specific phobias can impose on people’s lives, only about 25% seek treatment, with psychotherapies and long-term pharmacotherapy often failing to produce lasting results, and traditional ETs being ineffective for 35% of those with fear related disorders (52, 91, 92). For instance, propranolol is commonly used to treat physiological symptoms of anxiety and stress-related disorders as a non-cardioselective beta-blocker, that readily crosses the blood-brain barrier to block beta adrenergic receptors and simultaneously stimulate serotonin receptors (94–97). However, one study found that despite long term benefits in phobic reactions to spiders, reductions in Spider Phobia Questionnaire (SPQ) scores did not significantly decrease until 3 months after treatment (95). Another study found propranolol delivered post retrieval of emotional memories attenuated fear potentiated startle (FPS) responses with memory reinstatement sensitivity not significantly differing from the controls group, concluding that propranolol may alter fear expression without providing adequate cognitive alterations (98). Others have found propranolol has no significant effects on reducing FPS, implying that propranolol may not cause physiological alterations on extinction learning at all (99).

The first line short-term treatment for anxiety, benzodiazepines, entail risk for developing tolerance, dependence, and sedative states that can interfere with healthy functioning (100). The recommended long-term pharmacological intervention for anxiety disorders are selective serotonin reuptake inhibitors (SSRIs), an antidepressant drug. However, SSRIs inhibit MDMA’s subjective drug effects (1), and phase 2 MDMA trials have shown considerably lower dropout rates (6.8%) than those receiving sertraline (28%) and paroxetine (11.7%) treatment; perhaps due to the direct supervision and low number of drug sessions involved in MDMA-AT (42).

## 3 The biopsychosocial model in application: MDMA-assisted Dyadic One Session Treatment (DOST) for spider phobia

**Figure 2:**
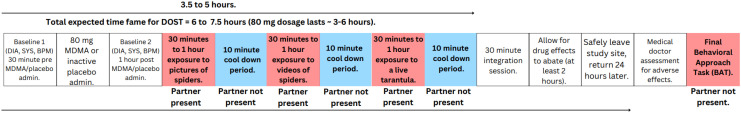
Experimental model for Dyadic One Session Treatment (DOST) for spider phobia. After undergoing baseline physiological measurements and administration of MDMA or placebo, targets will engage in a structured hierarchy of exposure to phobic relevant stimuli, proceeding to the next level of exposure after 30 minutes if their peak SUDs have decreased by 50%, and no longer than 1 hour if this level of habituation has not occurred. Following the completion of DOST protocol, integration session, and discharge from the exposure site, targets will return to the exposure site for a final medical assessment and behavioral approach task (BAT) to assess for any effects DOST protocol had on fear extinction.

With a preliminary understanding of the neurobiological mechanisms and acute psychological effects of MDMA usage, and the nature of specific phobias, we introduce MDMA assisted Dyadic One Session Treatment (DOST) for specific phobia, to exemplify the biopsychosocial model of MDMA-AT to be tested in application. We propose testing how MDMA administration, and a dyadic model, may optimize exposure to phobic stimuli in a single session of graded exposure that more accurately models MDMA assisted fear extinction paradigms typically used in studies to date (4, 79), instead targeting the preconditioned fear of spiders rather than laboratory conditioned fear. The MDMA-assisted DOST protocol is adapted from Öst’s (101) one session treatment (OST) of specific phobias, with controlled studies yielding positive response rates of at least 76% (102). As a cognitive behavioral treatment, OST is believed to target the physiological, behavioral, and cognitive facets of phobic responses (102), which may naturally pair well with MDMA’s hypothesized biopsychosocial therapeutic mechanisms. In the DOST model, those seeking treatment (the “target”) arrive at the exposure site with a “partner,” a highly trusted individual from their personal life, to accompany them throughout each level of exposure. According to original OST guidelines, the total treatment lasts roughly 3 hours, with no set timeline for exposure to each stimulus in the hierarchy, with Öst recommending clients move onto the next level of the exposure after they have effectively habituated to the current exposure stimuli (102). Thus, we recommend DOST targets should be exposed to a hierarchy of increasingly intense and realistic phobic relevant stimuli for at least 30 minutes before moving to the next stage of the hierarchy if their subjective unites of distress (SUDs) have decreased by 50% of peak levels, and up to 1 hour maximum if that degree of habituation has not occurred. Here, we recommend a general guideline of the exposure level process to the specific phobia of spiders (1): pictures of spiders (2), videos of spiders, and (3) a live tarantula (**Figure 2**). However, those conducting DOST exposure protocol should work collaboratively with targets to develop a hierarchy that is appropriate for their fear level and form of specific phobia, as per general cognitive behavioral techniques and as recommended in original OST guidelines (102). Although imaginal exposure is used in METEMP studies for PTSD, PTSD often entails narratives from the traumatic event(s) that are cognitively attached to fear inducing stimuli, which makes it a natural pair for MDMA assisted cognitive processing via imaginal exposure. However, such narrative driven cognitions may not be present in many forms of specific phobias. Therefore, we do not include imaginal exposure in our general recommendations for the DOST protocol. However, therapists/researchers should collaborate with targets to determine if imaginal exposure is appropriate to include in the phobic hierarchy according to each individuals’ level of fear, etiology of phobic associations, and willingness to engage in exposure exercises.

**Figure 3:**
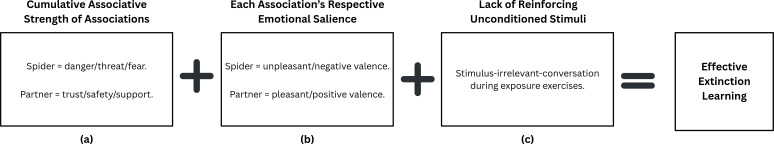
Proposed inhibitory learning model for MDMA-assisted Dyadic One Session Treatment (DOST) for spider phobia informed by Rescorla and Wagner’s (22) Error Correction Model of extinction learning in which **(a)** cumulative associative strength of all present stimuli, along with **(b)** each stimuli’s own saliences, and **(c)** without reinforcement of the unconditioned stimulus (US) determines the degree of extinction learning.

The decision to include partners and employ a dyadic model in exposure exercises is two-fold. First, a trusted partner allows for a naturally engaging source of exposure-irrelevant target focus that can be utilized to support adaptive exposure engagement and maximize emotional processing and inhibitory learning. This is supported by findings from a study in which 27 spider phobic individuals underwent three 10-minute *in-vivo* exposure sessions to a live spider. These individuals showed larger reductions in fear responses, as measured by the FSQ, BATs, and SUDs, when exposures occurred in the presence of another individual while discussing topics unrelated to spiders during exposures vs. discussing aspects of the current spider exposure (103). It is speculated that phobic-stimulus-irrelevant conversation can provide a useful distraction to de-escalate those with overwhelming and therapeutically resistant levels of fear activation in such *in-vivo* exposures (18). Second, the decision to include partners (vs. unknown others like experimental or clinical staff who might serve as conversants with the target) in exposure exercises is supported by rodent studies using PTSD-behavior-like fear conditioning paradigms showing significantly improved fear extinction outcomes with inclusion of non-trauma exposed conspecifics during extinction exercises (104–108). Moreover, social support cues can significantly inhibit initial and long-term fear responses in humans (109). These findings oppose the function of stimuli individuals engage with to inhibit initial conditioned fear reactions, called safety signals, that can be detrimental to long term fear reductions (16), causing some to argue that social support is more appropriately considered a prepared fear suppressor, rather than a safety signal (109). For instance, presenting faces of loved ones with fear cues during fear conditioning did not lead to fear responses when the faces were absent, with mental imagery of loved ones yielding similar results, and other research showing images of socially supportive individuals lead to greater reductions in fear than those of strangers or neutral figures (109). It is also well known that social support can help facilitate the processing of stressful situations from a psychological and physiological basis (109). Thus, it is posited that social support may cue individuals to supportive resources when facing threat, reducing the perceived aversiveness of the stimuli, without diminishing the expectation of such stressful experience to occur in the future (109). Safety signals only diminish expectations of threat occurrence, which can be detrimental to long term fear extinction despite initial reductions in fear responses (109). It is also hypothesized that social support in the presence distressing stimuli engages the opioid system that reinforces social intimacy, alongside the opioid system that helps process fear and pain, leading to long term reductions in fear, although more research is needed in this area (109). Regardless, social support may be a useful, pragmatic, noninvasive augmentation for exposure therapies (109). Thus, the familiarity and fondness of the target with their partner as well as the therapeutic instruction to focus conversational engagement on topics unrelated to the current exposure exercise (e.g., talking about plans for the upcoming weekend vs. how scary/unpleasant it is to be looking at pictures of spiders) is expected to therapeutically maximize on such demonstrated experimental phenomena. Finally, the prosocial and empathogenic qualities of MDMA are expected to further synergize with the conversational focus and interpersonal engagement of the target with the trusted other to further drive feelings of safety and trust in the target in the context of an otherwise fear-conditioned stimulus/situation (spider-related stimuli), which is theorized to further enhance fear extinction and extinction retention. In line with inhibitory learning informed by the error correction model (**Figure 3**), the combined strength of discrepant associations during exposure, in this case, feared stimuli (i.e., spider-danger/fear/threat) and the partner (i.e., partner-safety/trust/support), with each of their respective emotional saliences (i.e., spider-unpleasant valence and partner-pleasant valence), without reinforcing the US (stimulus-irrelevant-conversation) may lead to enhanced fear extinction and naturally pair well with the pharmacological and prosocial effects of MDMA.

Between each level of exposure, partners will be asked to step out of the room for a 10-minute break or “cool down period,” before returning for the next stage of the exposure hierarchy. The 10-minute cool down periods (where the partner is absent) serve functional purposes, in which the primary therapist delivering DOST may step out of the exposure room if need be (i.e., bathroom or lunch break), allowing for the assistant therapist to step in and continue facilitation of the exposure protocol. The 10-minute cool down periods would also provide more than adequate time to set up for the next stage of the exposure protocol.

Although the optimal MDMA dosage will require empirical study, for targets undergoing MDMA assisted DOST, it makes sense to start investigation with 80 mg of MDMA, the minimum therapeutic dosage based upon existing research (110). Baseline physiological measurements for those receiving MDMA can be recorded 30 minutes prior and 1-hour post administration including systolic (SYS) and diastolic (DIA) blood pressure, and heart beats per minute (BPM), which should be recorded throughout DOST protocol (consistent with current best practices for current MDMA therapeutic administration). Elevated blood pressure is consistent across MDMA studies with human subjects (45, 79) so is not a cause for concern unless it reaches dangerously high levels or remains elevated after the acute subjective effects have abated.

The time starting from initial baseline physiological measurements through the end of the graded exposure process 3.5–5 hours, and like previous research, will capitalize upon knowledge of MDMA’s time course of subjective effects to coincide with the treatment’s therapeutic schedule (30). Previous literature details how the subjective effects of MDMA typically last between 3–6 hours (36, 111). The DOST protocol is designed so that initiation of exposure level 1 takes place when subjective drug effects begin to near peak levels (111). As exposure levels increase in intensity and realism, so will the drug effects increase in intensity. This way, participants undergo more acute effects of MDMA that may increase engagement in exposure exercises, as the exposures entail increasing difficulty of engagement without pharmacological augmentation. By the time targets reach the final level of exposure after about 2.5-3.5 hours post MDMA administration, the drug effects will still be near peak levels while on a downward trajectory (111). Participants should be monitored and allowed to rest or interact with their partner for at least 2 hours following the end of the DOST protocol to allow the subjective drug effects to completely dissipate. Moreover, the roughly 3-hour exposure timeframe is in line with what is commonly practiced in ET sessions for specific phobia (91).

24 hours after completion of the DOST protocol, targets should return to the exposure site for a structured behavioral approach task (BAT) to a live tarantula to assess for fear extinction retention from the DOST exposure. This BAT has been significantly correlated to neurophysiological spider phobic fear responses (93). Following the DOST protocol and BAT task, participants should complete self-report outcome measures such as the Spider Phobia Questionnaire (SPQ) and Fear of Spider Questionnaire (FSQ) in a handful of follow up assessments to measure/track any long-term decreases in spider phobic symptomatology. Both the SPQ and FSQ are empirically validated to discern non-phobic and phobic individuals, are sensitive to alterations in phobia from treatment, with adequate test-retest reliability (112).

### 3.1 MDMA-assisted DOST rationale

The goal of treatment for specific phobias is to decrease fear, decrease avoidance behaviors, and reduce impairment from distressing phobic reactions, with ETs being the most studied and efficacious treatment option, typically involving repeated sessions, usually lasting between two to three hours, being gradually exposed to increasingly feared stimuli until fear responses completely abate (13, 91). This kind of treatment is referred to as graded exposure. Its efficacy in treating anxiety disorders is well documented (113), and it is a standard treatment for specific phobias (113). Meta-analyses have revealed that multisession treatments of exposure only slightly outperform single session treatments regardless of the specific phobia targeted (13). Moreover, roughly 25% of phobic individuals do not seek exposure-based treatment, due to intense fear of confronting phobic stimuli (13). Thus, it has been pustulated that gradual exposure techniques may help mitigate challenges that contribute to lack of treatment seeking behaviors (114), which we include in the DOST protocol, rather than more strictly *in-vivo* exposures outlined in traditional OST exposures.

### 3.2 Hypothesized mechanisms of action

We hypothesize that MDMA’s neurobiological effects will help optimize participant engagement in the DOST protocol and facilitate adaptive reassociations of phobic reactions. We believe that MDMA’s putative inhibitory effects on amygdala and hippocampal function and promotion of increased amygdala-hippocampal connectivity will allow for enhanced extinction of conditioned fear responses to spiders and promote enhanced extinction retention, thereby reducing subsequent negative affective reactions to spider-related stimuli encountered in day-to-day life. Moreover, we believe that MDMA’s hypothesized acute effect of attenuating insular activity will desensitize visceral physiological thresholds of tolerance, widening the Window of Tolerance to distressing physiological reactions that may occur in confronting phobic stimuli. We also posit that MDMA-promoted increases in synaptic serotonin release and concomitant effects on oxytocin will lead to increased positive affect and heightened levels of sensitivity to social reinforcers/social rewards (115, 116), which is here theorized to be engaged by structured social interactions with the trusted partner, two factors that may furthermore serve to enhance fear extinction learning and retention (117, 118). The vmPFC and ACC, in particular, have been found to mediate the effects of social buffering on fear extinction (117) as well as implicit forms of emotional regulation (119). This confluence of effects is expected to decrease avoidance behaviors and facilitate long term memory storage of fear extinction learning. This upregulation in serotonin, oxytocin, and vmPFC activity is also hypothesized to engender more positive affect and prosocial behavior that could pair well with the dyadic model, potentially aiding adaptive emotional processing to phobic stimuli during DOST protocol. Cumulatively, these neurobiological mechanisms may result in increased capacity for and retention of fear extinction, enhanced approach behavior, and decreased phobic symptomatology.

### 3.3 MDMA-assisted DOST protocol

#### 3.3.1 Study site and dyads

Consistent with existing best practices, MDMA would be administered in a safe, soothing environment. This is partially attained through a highly trusted individual that the target can bring with them to the exposure site for accompaniment during exposure exercises. The partner may be a close friend, family member, romantic partner, etc. However, it would seem important that the partner be someone with whom the target can feel safe and share trust to maintain strong rapport within the dyad. While undergoing physiological baseline measurements, and in the 1-hour post MDMA administration, targets will spend time in a comfortable setting marked by dim lighting, a cushioned chair to sit in, some wall decorations, and soothing music to listen to with their partner in the presence of the primary therapist/researcher. Unlike many MDMA-AT studies, targets will not be provided blindfolds or ear coverings, as these will not be used as part of the exposure protocol. During the exposures, dyads are instructed to talk about anything they would like with the exception of phobia-relevant stimuli or thoughts/feelings around the exposure process they are engaging in, if the target’s visual attention is allocated toward the phobic stimuli. To maximize likelihood of a positive therapeutic experience for the target, the partner, themselves, should not have any substantial or impairing level of spider phobic fears/behaviors.

Also, touch should be held to a minimum on the target from therapists, researchers, or partners, so as to not distract from optimal visual attention with the phobic stimuli. However, considering the entactogenic and prosocial effects of MDMA, partners may engage in gentle touch with the target (i.e., a hand on the shoulder), after obtaining the target’s explicit consent, only if the phobic stimuli is so intense for the target, that they cannot engage with the exposure exercise. Such minimal, gentle, and consensual touch may prove to be a grounding experience for the target, so long as it does not distract them from the exposure stimuli.

#### 3.3.2 Pre- DOST

The treatment begins with a psychoeducation phase. In it, the dyad should be debriefed in detail of the entire process of DOST protocol prior to initiating the exposure process, including drug dosage, the exposure stimuli, exposure level length, length of entire DOST protocol, and how long the drug effects should last. The dyad should be informed that the DOST protocol is based upon a naturalistic biopsychosocial model and is purposely designed to optimize treatment based on the prosocial and neurobiological effects of MDMA. The dyad should also be informed that as part of the protocol, they are required to return to the exposure site 24 hours later to assess for any unintended side effects from the MDMA and to engage in follow up BAT. The dyad will then engage in rapport building conversation with the therapist to develop trust with the individual/individuals that will be facilitating the DOST protocol. The rapport building session will also include basic psychoeducation regarding both the benefits and potential side effects of MDMA administration for therapeutic purposes (40, 41, 120–127).

#### 3.3.3 DOST protocol

The dyad should be reminded of the procedure for each exposure level before initiation of each exposure level. SUD scores should be inquired by the researcher after every 5 minutes on a 0–100 scale in line with protocol described in previous literature (128). 0 would indicate no fear and 100 would indicate the most fear that could possibly be felt. Below, the general recommended exposure hierarchy for DOST of spider phobia is outlined, but future researchers should work collaboratively with targets to determine an individualized phobic hierarchy should such a protocol receive funding and regulatory approval.

Exposure level 1 involves exposure to physical or digital pictures of various spiders both harmless and potentially harmful to humans. Researchers may use protocol from previous research using 60 spider phobic images to elicit threat based physiological arousal (19). This includes maintaining continuity between images by including a single spider centered in each slide, with backgrounds using the same color in each picture, changing to the next picture every 4 seconds. Such protocol has been shown to be sensitive to subjective ratings of distress between specific phobia stimuli (i.e., spiders vs. snakes) and phobic versus positive (i.e. puppies) and neutral images (19). Targets may engage in stimulus irrelevant conversation with their partners throughout this exposure period as long as visual attention is maintained with the pictures. If visual contact with the stimuli is visibly broken, the therapist will ask the target to redirect their visual gaze to the phobic stimuli.

Exposure level 2 involves watching videos of spiders. Targets may engage in stimulus irrelevant conversation with their partners throughout this exposure period as long as visual attention is maintained with the videos.

Exposure level 3 involves the target viewing a live tarantula, presented by the researcher in a small glass container with a removable lid on a pushcart. The researcher should clearly and calmly inform the target of every step they take to reveal the tarantula, to not surprise the target. In line with exposure procedures to live spiders from Johnstone and Page (103), targets will be instructed to keep their chin about 20 cm above the center of the container, looking down at the live tarantula, being encouraged to maintain visual attention to the stimulus. Once the target is properly positioned to view the tarantula, they can begin stimulus-irrelevant-conversation with their partner.

#### 3.3.4 Post-DOST

After all 3 levels of DOST are completed, the target will engage in the final cool down period. Upon completion, the researcher should inform the target that the DOST procedure has been completed and congratulate them for their efforts. The researcher will then engage in a brief integration session to help the target emotionally process the DOST protocol. The integration session should be directed by the target, lasting no longer than 30 minutes. Upon integration session completion, the target will be allowed to rest with their partner while the primary therapist/researcher is present, until at least 5 hours have passed since MDMA administration, and at least 2 hours post integration session (41). Once 5 hours have passed, the drug effects have completely dissipated, and the target is in a safe and stable condition (including normalized heart rate and blood pressure), the researcher can allow them to leave the exposure site with the accompaniment of their partner, in line with protocol from Imperial College London (41).

#### 3.3.5 Follow up BAT

24 hours later, dyads should return to the exposure site, and targets should be evaluated for any adverse events from MDMA. Upon successful completion of this exam, targets will engage in a final BAT to a live tarantula to assess how well MDMA assisted DOST aided targets’ fear extinction learning. The partner will be absent for this task to assess for fear extinction in a somewhat novel setting. Participants will sit in a comfortable chair at the far end of the exposure space. The researcher will inform the target they are briefly leaving to get the tarantula and then announce their presence before re-entering the exposure room with a pushcart carrying a transparent terrarium covered by a towel that contains the tarantula. The BAT continues in a 7-step process, each step lasting 2 minutes. The researcher should ask the target to report their SUDs at the very beginning of steps 1 through 5 of the BAT, and immediately after touching the tarantula with a pencil for step 6 and their finger for step 7. Each BAT step is outlined below (93).

BAT Step 1) The researcher instructs the participant to rise from their chair and move one yard directly towards the covered terrarium.
BAT Step 2) The researcher instructs the participant to move another yard directly towards the covered terrarium.
BAT Step 3) The researcher instructs the participant to fully approach the terrarium.
BAT Step 4) The researcher instructs the participant to remove the towel from the terrarium and look directly at the tarantula.
BAT Step 5) The researcher instructs the participant to open the terrarium.
BAT Step 6) The researcher instructs the participant to touch the tarantula with an unsharpened pencil.
BAT Step 7) The researcher instructs the participant to gently touch the tarantula with their index finger.

#### 3.3.6 Hypothesized primary outcomes

We hypothesize that utilizing OST, adding a dyadic component, and augmenting with MDMA will result in greater exposure engagement, greater rates of completion to follow up BAT, and larger magnitude longitudinal decreases in spider phobia symptomatology compared to an active or inactive placebo comparator condition as well as MDMA-assisted OST without a partner.

#### 3.3.7 Testing and validation

**Figure 4:**
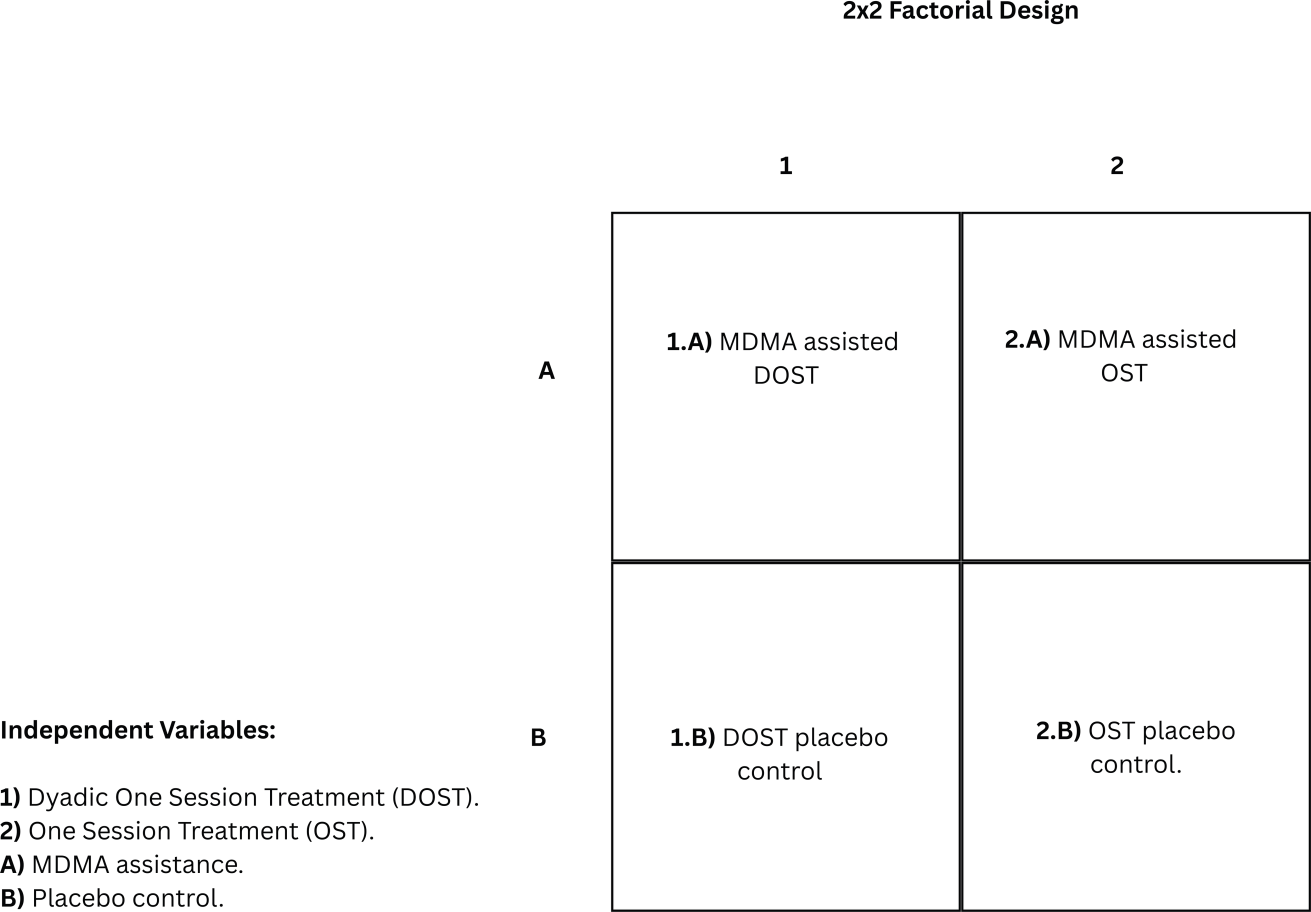
2x2 factorial design of independent variables to experimentally test the efficacy of Dyadic One Session Treatment (DOST) compared to One Session Treatment (OST) for specific phobia with and without MDMA augmentation or a placebo.

Here, a 2x2 factorial design (**Figure 4**) is proposed to test the efficacy of MDMA-assisted DOST against an active/inactive placebo and individual OST. The two independent variable categories considered are (a) MDMA versus placebo administration, and (b) dyadic exposure versus individual exposure. One group undergoes MDMA-assisted DOST protocol, with another receiving MDMA-assisted individual OST. The inactive placebo control groups should be organized in the same fashion. The 10-minute breaks in which partners are absent can also be used to assess how partners may serve as a counterconditioning stimulus of the opposite valence to the phobic stimuli (118) when only present for exposure exercises to further facilitate fear extinction processes using the 2x2 factorial design.

The dependent variables proposed are completion of graded exposure levels, completion of 24 hour follow up BAT, and longitudinal reductions in spider phobia symptomatology. Spider phobic symptomatology can be measured by administering the SPQ and the FSQ at various time points after completion of the exposure protocol. We propose targets complete the SPQ and FSQ 1 week, 2 weeks, 1 month, 2 months, 3 months, and 4 months post graded exposure and BAT task. As such, between group differences in reductions of spider phobia symptomatology can be tracked longitudinally in a more frequent fashion than what is currently seen in MDMA-AT clinical trials.

### 3.4 Contributions to the field

There are currently no studies testing MDMA’s therapeutic potential to treat specific phobias, with MDMA-AT clinical trials primarily targeting PTSD (30, 31, 110, 129–133).This initial focus is warranted, given only about half of individuals develop clinically significant improvements from gold standard treatments for PTSD, and these treatments are characterized by high dropout rates (134). Considering the current limitations of anxiety and stress disorder treatments; it is imperative to develop novel treatment approaches from an evidence based and mechanistic perspective that optimally target the differential ways similar emotional structures appear across disorders.

Findings from testing the DOST protocol on a relatively uncomplicated pure fear-based disorder, such as specific phobia, can be used to inform novel treatments for various anxiety and stress disorders like PTSD that all involve maladaptive alterations in fear-responsive neural circuits. DOST can benefit the field by attempting to capitalize on the pharmacological mechanisms of MDMA with a learning theory-informed cognitive behavioral approach. Traditional psychedelic-assisted therapies use non-directive supportive approaches, which may be suboptimal (135). This has spurred some to recommend using evidence based cognitive-behavioral procedures, such as ET that target decreasing experiential avoidance (1, 5, 43, 135). DOST’s naturalistic design informed by the biopsychosocial model of MDMA-AT attempts to synergize acute drug effects with evidence-based therapeutic approaches to optimize treatment outcomes.

Although PTSD involves prominent fear-based symptoms, it is also characterized by a heterogenous array of challenging emotions like anger, grief, shame, guilt, and sadness (24, 109) that may impede optimal therapeutic outcomes from capitalizing on ETs in the context of MDMA administration. By investigating MDMA’s ability to treat more emotionally uniform pathologies predicated on fear, such as specific phobia, the field may benefit from improved knowledge regarding how MDMA can accelerate fear extinction-based therapeutic approaches and how these might be employed as a treatment strategy across anxiety, stress, and fear-based disorders. Moreover, research on dyadic MDMA-ATs and CBCT for PTSD have lacked consistent use of active control groups, making the proposed 2x2 factorial design a proper fit to fill the gap in the current literature, i.e. to directly compare dyadic models to individual models with and without MDMA administration.

## 4 Discussion

Here, we present a novel MDMA exposure protocol hypothesized to facilitate treatment of spider phobia that is informed by a biopsychosocial model of MDMA-AT. We have discussed the theoretical structures for the biopsychosocial model, the neurobiological mechanisms underlying MDMA and fear extinction, the current understanding of specific phobias, and the limitations for current treatments. We have used this understanding to propose the DOST model for testing and validation. One feature of importance to the DOST model is that it is designed to optimally capitalize on the acute effects of MDMA for therapeutic use. Second, by targeting specific phobias as a natural experimental model of conditioned fear and avoidance, we can more precisely understand the potential for MDMA-AT to treat fear-based disorders. Third, a 2x2 factorial design is proposed to understand if longitudinal fear extinction can be more readily promoted using a dyadic MDMA model, compared to those receiving individual treatment with and without pharmacological aid.

### 4.1 Limitations

The most glaring limitation for the DOST model is that there are no current studies assessing the potential therapeutic benefits of MDMA on specific phobias. Thus, despite MDMA’s known effects on fear extinction and fear-related neurocircuitry, there is no quantitative data to support the use of MDMA-AT for treatment of specific phobias. Also, since the graded exposure is condensed into one session, it is possible that those receiving the active placebo will not derive significant beneficial gain, as graded exposure is traditionally conducted across multiple sessions with single session treatments typically reserved for flooding techniques involving initial exposure to the most intense feared stimuli possible (136). However, positive results from the proposed study design could be extremely informative regarding MDMA’s ability to consolidate treatment schedules. Furthermore, there is limited research on dyadic models of MDMA-AT, with those discussed containing no active placebo control group. This renders it difficult to determine how beneficial dyadic MDMA-ATs are compared to non-pharmacological and individual models. Moreover, on the dyadic component, requiring the presence of a partner not receiving treatment for a several hour exposure session would likely limit the accessibility of the treatment approach, as researchers and clinicians then must account for finding availability that works for more than one participant at a time.

The DOST model also inherits limitations from research on its predecessor. Although the theory behind Öst’s OST protocol includes one of the most optimally comprehensive biopsychosocial understandings of pathological fear, OST only meets “probably efficacious status” due to small sample sizes and lack of assessment against gold standard empirically supported ETs (102). However, OST effect sizes are large for wait-list controls and uncontrolled studies, with small to moderate effect sizes in the minority of studies using active controls groups, and 85%-90% improvement rates for adults (102).

Although the presence of a partner is meant to capitalize on the prosocial effects of MDMA, partners may serve as a cognitive distraction for the target. Although distraction in exposure treatment for spider phobia has positive preliminary findings (103), the effectiveness of distraction during exposure is historically understudied, with the effectiveness of distraction during fear provoking situations being unclear (137). Furthermore, a systematic review has found that distraction may hinder extinction learning in ET for specific phobias (114). Foa and Kozak (14) have also theoretically argued that distraction can interfere with emotional processing by obfuscating acquisition of new information into memory and impeding elicitation of genuine fear responses. Although, inhibitory learning theories have prevailed over the notion that full fear responses are integral to extinction learning (16), and consistent positive results of rodent studies show the presence of a neutral conspecific during extinction learning improves fear extinction. Yet, such rodent studies do not capture the differential interpersonal roles that humans have which contribute to individual differences in stress responses. Moreover, despite positive preliminary findings indicating social support cues may serve as prepared fear suppressors, this model also has its limitations. For instance, human studies in this realm suffer from small sample sizes, it is not fully understood how individuals become socially supportive figures, and such studies have not been tested on clinical populations (109). The current hypothesis is that the presence of and interaction with a trusted partner during exposure may serve as an intrinsic reward component of the experience and capitalize on counterconditioning processes to enhance extinction and inhibitory learning, though whether this would be beneficial or detrimental (e.g., viewed as a distraction rather than a rewarding experience) remains to be tested. There are also potential gender differences that could impact outcomes related to dyadic exposure therapy. For instance, shared experiences of stressful events in the daily lives of romantic partners result in less stress for 99% of women, compared to only 42% of men (138). The 2x2 design proposed will allow for further understanding of the effects of the dyadic interaction during ET, specifically the comparison of the DOST procedure vs individual OST both without MDMA augmentation.

### 4.2 Future directions

The biopsychosocial model proposed should be interpreted as preliminary and in need of additional development and refinement. Like many psychedelic compounds, there is more to be discovered and understood about MDMA’s mechanisms of change that contribute to its therapeutic effects. The aim of this model is to consolidate the current understanding of MDMA’s influences on the mind and body, outward social behavior, and opportunities for cognitive adaptation. As understanding develops, so should the biopsychosocial model also develop to most accurately portray the cumulative effects MDMA has on human experience.

Furthermore, the DOST protocol herein is used to highlight how the biopsychosocial model can inform MDMA-ATs by targeting chronic fear in specific phobias, which was chosen due to MDMA’s effects on fear extinction and fear-responsive neurocircuitry. Future researchers may use the proposed DOST protocol and 2x2 factorial design to pilot clinical trials that examine its efficacy with and without the augmentation of MDMA, with follow up assessments such as the SPQ and FSQ to determine the long-term effects of the exposure protocol. However, considering the limitations in understanding of the development of socially supportive individuals (109), researchers should conduct prescreening of targets’ partners to determine if their support will be of a positive nature and not be of detriment to the target during the exposure protocol. Such screenings may include questions regarding prior instances in which partners have supported targets through stressful situations. This way, researchers can attempt to control for distractions or negative impact of well-intended support that partners may provide targets. Researchers should also carefully analyze partners’ support styles during and after the DOST protocol to refine understanding of what behaviors and lines of conversation are supportive and facilitate engagement to exposure stimuli. Researchers should also consider piloting DOST studies without MDMA augmentation compared to OST and/or already established gold standard ETs before implementing an MDMA component; to further understand the effects social support has on inhibitory and extinction learning. Considering discrepant findings on the effectiveness of shared stressful experiences between men and women (138), researchers may also consider comparing DOST outcomes across genders. If such proposed studies yield positive results and lead to MDMA-assisted DOST clinical trials, findings may inform MDMA-ATs for other anxiety and stress-related disorders. Although MDMA’s empathogenic qualities may help alleviate emotions such as guilt and shame associated with PTSD, positive findings from focusing more granularly on MDMA’s effects on fear extinction may help advance understanding of how other emotional structures can be targeted and treated that are present in more complex disorders such as PSTD and SAD.

In summary, we propose that jointly conceptualizing the effects psychedelics have on human experience across biological, psychological, and social domains can best inform strategies for optimizing therapeutic effects ethically and safely. These efforts may also lead to a deeper understanding of the best therapeutic modalities to be assisted and pinpoint which psychedelic compounds and with what behavioral treatment modalities are most advantageous to treat various psychological diagnoses.

## Data Availability

The original contributions presented in the study are included in the article/supplementary material. Further inquiries can be directed to the corresponding author.
